# A Comparative Evaluation of Surface Roughness and Shear Bond Strength of Two Ceramic Repair Systems After Different Chemical and Mechanical Surface Treatments: An In Vitro Study

**DOI:** 10.7759/cureus.117126

**Published:** 2026-09-29

**Authors:** Priyanka A Patil, Shubha Joshi, Pronob Sanyal

**Affiliations:** 1 Department of Prosthodontics, School of Dental Sciences, Krishna Vishwa Vidyapeeth (Deemed to be University), Karad, IND

**Keywords:** acidulated phosphate fluoride, ceramic repair, phosphoric acid, shear bond strength, universal testing machine

## Abstract

Background

Porcelain fracture in metal-ceramic restorations remains a common clinical complication. Effective intraoral repair depends on durable adhesion between the repair material and the fractured substrate. Surface treatment methods significantly influence the mechanical and adhesive properties of these repairs, yet the comparative efficacy of newer approaches, such as glycine powder air abrasion, remains insufficiently explored. This study aimed to evaluate and compare the effects of four different surface treatments: a diamond bur, air-abrasive glycine powder, 1.23% acidulated phosphate fluoride (APF) gel, and 37% phosphoric acid, on the maximum load, shear bond strength, and failure modes of repaired metal-ceramic surfaces using two commercially available ceramic repair systems.

Materials and methods

Sixty-four nickel-chromium metal-ceramic specimens were evaluated. The specimens were divided into two groups (n = 32 each) according to the ceramic repair system used: Group A (Ivoclar Vivadent) and Group B (Shofu). Each group was further divided into four subgroups (n = 8) based on the surface-treatment modality. Surface defects were standardized and repaired according to the manufacturers’ protocols. Specimens were stored in distilled water at 37°C for seven days and thermocycled for 500 cycles between 6°C and 60°C. Shear bond strength was tested using a universal testing machine. Failure modes were examined under a stereomicroscope. Data were analyzed using one-way analysis of variance (ANOVA), followed by Tukey’s post hoc test for multiple comparisons where appropriate. Failure-mode distributions were analyzed using the Fisher-Freeman-Halton exact test (α = 0.05).

Results

In both groups, glycine powder produced the highest mean maximum load and shear bond strength, whereas phosphoric acid yielded the lowest values. Significant differences were observed for maximum load in both groups and for shear bond strength in Group A. Failure mode analysis showed fewer adhesive failures with glycine powder and APF gel.

Conclusions

Surface treatment with glycine powder effectively enhanced maximum load and shear bond strength and may provide clinical benefit for intraoral repair of metal-ceramic restorations.

## Introduction

Porcelain-fused-to-metal (PFM) restorations have long been a standard in fixed prosthodontics because the metal substructure offers high strength and durability, combined with the aesthetic advantages of a porcelain veneer [1]. Despite the widespread use of dental ceramics, including porcelain, these materials exhibit inherent limitations. Feldspathic porcelain, commonly used in such restorations, has a compressive strength approximately 2.5 times lower than natural enamel [2]. Consequently, porcelain components are susceptible to fracture under various conditions. These include impact loading, functional occlusal forces, thermal expansion mismatch between the porcelain and the metallic substructure, selection of metal alloys with a low elastic modulus, excessive seating force during trial fitting or cementation, suboptimal prosthesis design, intrinsic material defects, and external trauma [3]. In addition to mechanical stresses, adhesive interfaces are exposed to various intraoral challenges, including fluctuations in temperature, salivary exposure, pH variability, and cyclic loading.

These factors can compromise the ceramic-metal bond, particularly due to the inherently low tensile strength of ceramic materials. As a result, porcelain failures are relatively common, with reported incidence rates ranging from 2.3% to 8% [4,5]. Such failures are recognized as the second most prevalent cause of restoration failure, following secondary caries [6,7]. Porcelain fractures can be categorized based on their severity and structural involvement: simple fractures involve only the ceramic body; mixed fractures expose both ceramic and metal components; and complex fractures result in extensive metal exposure.

The repair of fractured metal-ceramic restorations requires restoration of both the functional and esthetic properties of the prosthesis through the application of various repair materials [8,9]. The establishment of reliable and durable chemical bonds between dental ceramics and repair materials is of paramount importance. Achieving effective adhesion between these materials and the substrate to be repaired necessitates appropriate surface conditioning [10]. Such surface treatments may enhance mechanical retention, including techniques such as airborne particle abrasion with aluminum oxide, rotary instrumentation using diamond burs, and etching with agents like hydrofluoric acid, phosphoric acid, or acidulated phosphate fluoride (APF) [11-14].

In addition to mechanical methods, chemical adhesion can be promoted through the application of silane coupling agents and adhesive primers [11,15-18]. Some approaches integrate both mechanical and chemical mechanisms to optimize bond strength. These include silica deposition via conventional airborne-particle abrasion [8,19-21]. Often, a combination of these surface treatments is employed to enhance the bond strength between the repair material and the fractured substrate. Air abrasion improves the strength of bonds in intraoral repair systems by creating a very fine, rough surface with obtuse angles. This roughness increases the total surface area, reduces surface tension, and helps the resin adhere better by improving wetting [22].

In this study, we have used air-abrasive glycine powder for air abrasion as an innovative method for surface treatment to create surface roughness on exposed metal-ceramic surfaces. Glycine powder is widely employed in the nonsurgical management of peri-implantitis, which entails the debridement and detoxification of implant surfaces within peri-implant pockets. Low-abrasive glycine, an amino acid-based powder, has been shown to effectively disrupt and remove biofilm without compromising the integrity of implant surfaces or the surrounding hard and soft periodontal tissues. Subgingival air polishing with glycine has demonstrated the ability to decontaminate periodontal pockets efficiently, causing minimal to no damage to root surfaces or adjacent soft tissues. Accordingly, glycine air polishing has been proposed as a preferable alternative to repeated mechanical debridement with conventional curettes during periodontal maintenance therapy [23,24]. The effect of glycine powder air abrasion on the surface topography of exposed metal-ceramic substrates requires evaluation, as the induced surface roughness plays a critical role in influencing the bond strength between the repair materials and the substrate.

This in vitro study focuses on the evaluation and comparison of the surface roughness created by four different surface treatments and the shear bond strength achieved following these surface treatments on exposed metal-ceramic surfaces repaired with two different commercially available ceramic repair systems (Ivoclar and Shofu). The chemical agents used in this study are 1.23% acidulated phosphate fluoride gel and 37% phosphoric acid, and the mechanical agents used are a diamond bur and glycine powder.

## Materials and methods

An a priori sample-size calculation was performed using G*Power software (version 3.1.9.7; Franz Faul, Universität Kiel, Germany). The calculation was based on a one-way analysis of variance (ANOVA), with an effect size (Cohen’s f) of 0.50, a significance level (α) of 0.05, and a statistical power of 80%. The analysis indicated a minimum required total sample size of 64 specimens. Accordingly, 64 nickel-chromium metal-ceramic specimens were included in the study, with eight specimens allocated to each of the eight experimental combinations comprising two ceramic repair systems and four surface-treatment modalities.

Sixty-four metal pellets of nickel-chrome alloy (NDN DFS alloy) were used to prepare study samples by ceramic build-up on one side of the pellet. For that, two coats of opaque porcelain were applied to a thickness of 0.2 millimeters and fired as per the manufacturer's instructions. A 0.8-mm dentin layer was applied, followed by a 0.8-mm enamel layer. Finally, the samples were completed and coated with glaze to ensure they had a consistent thickness of 2 millimeters (Figure 1). Samples were divided into two groups according to the ceramic repair system: Group A (Ivoclar, n = 32) and Group B (Shofu, n = 32). Each group was further subdivided into four subgroups (eight samples per subgroup) (A1-A4, B1-B4) based on the surface pretreatment method (Table 1).

**Figure 1:**
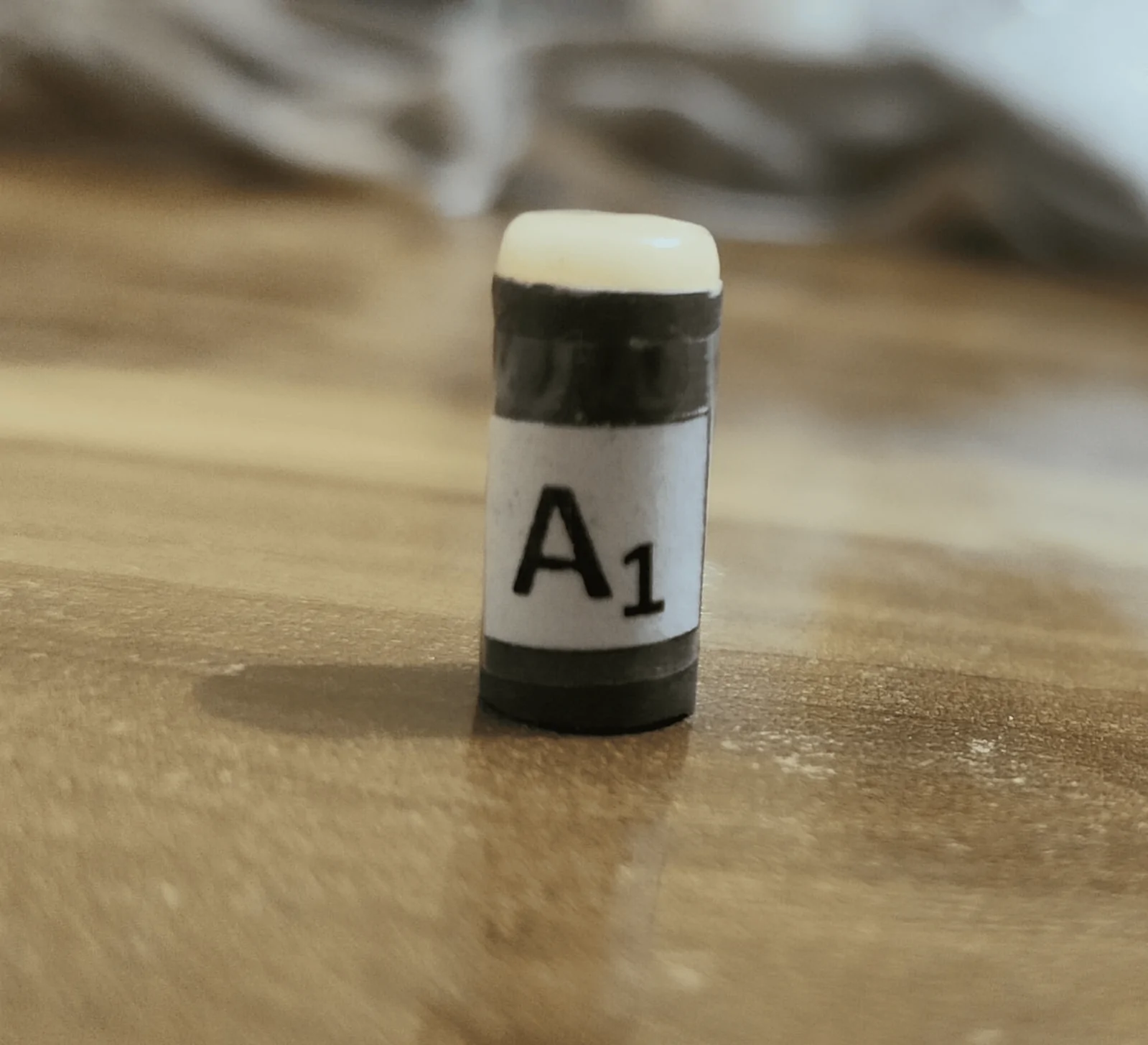
Preparation of samples by ceramic build-up of 2 mm on one side of the pellet (nickel-chrome alloy (NDN DFS alloy)

**Table 1:** Distribution of samples according to the repair system used and surface treatments

Type of surface treatment	Ivoclar repair system (Group A)	Shofu repair system (Group B)
Diamond bur	A1	B1
Air abrasive glycine powder	A2	B2
1.23% acidulated phosphate fluoride (APF)	A3	B3
37% phosphoric acid	A4	B4

Defects were created by removing ceramic with a diamond bur (ISO 199/018 TR-26) until metal exposure was achieved. After creating a surface defect, all samples were surface-treated to create a surface roughness. For A1 and B1 samples, a diamond bur (ISO 198/022 TR-14) was used; for A2 and B2 samples, glycine air abrasive powder (woodpecker air polishing powder (65um) glycine gentle) was used with a dental air polisher (Waldent Premium) at 4 bar pressure, 60° angulation, 10-mm distance for 10 seconds [25]. For A3 and B3 samples, 1.23% APF gel (Fluorotop APF) was used for six minutes [26], followed by rinsing and air-drying, and for A4 and B4 samples, 37% phosphoric acid (Waldent BEST Etch) for 60 seconds was used, followed by rinsing and air-drying.

Scanning electron microscopy (SEM) (Tescan VEGA 3) was performed to qualitatively evaluate and compare the surface morphology produced by the four surface treatments (Table 2). The available surface roughness values, expressed as arithmetic average roughness (Ra, µm), were summarized descriptively for each surface-treatment group. Because individual specimen-level Ra measurements were not available, measures of dispersion and inferential statistical comparisons could not be reliably performed. Therefore, the surface roughness findings were interpreted descriptively. After surface treatment, the defects on all samples were repaired with ceramic repair material. Samples from Group A were repaired with the Ivoclar ceramic repair system, and samples from Group B were repaired with the SHOFU ceramic repair system (Table 3).

**Table 2:** Surface treatments and surface roughness values APF: acidulated phosphate fluoride; Ra: arithmetic average surface roughness

Surface treatment	Surface roughness value (Ra, µm)
Diamond bur	138.766
Glycine powder	169.838
1.23% APF gel	147.043
37% phosphoric acid	72.336

**Table 3:** Stepwise protocol for metal ceramic defect repair using Ivoclar and Shofu ceramic repair systems

Step	Ivoclar repair system (Group A)	Shofu repair system (Group B)
1	Application of Monobond Plus with a microbrush on exposed ceramic and metal surfaces; allowed to react for 60 seconds; dried with oil-free air	Application of metal primer on exposed metal surface with a microbrush; left for 10 seconds
2	Application of Heliobond bonding agent with a microbrush on the repair area; excess removed with compressed air.	Application of opaque paste on exposed area; light-cured for 20 seconds
3	Light-curing of Heliobond for 10 seconds	Application of Ceraresin Bond 1; left for 10 seconds
4	Application of IPS Empress Direct opaquer on prepared metal surfaces (maximum thickness 0.5 mm)	Application of Ceraresin Bond 2; left for 10 seconds
5	Light-curing of the opaquer for 20 seconds	Light-curing for 20 seconds
6	Restoration with Tetric EvoCeram composite in ≤ 2 mm increments, adapted with a suitable instrument	Restoration with composite resin in increments
7	Light-curing of each composite increment individually for 10 seconds	Light-curing of each composite increment individually for 10 seconds

Specimens were stored in distilled water at 37°c for 7 days before thermocycling. After this, samples were thermocycled (6ºC: Make: LG, Model: 051SA; 60ºC: Mahavir, India) for 500 cycles between 6°C and 60°C with a 30-second dwell time. Specimens were mounted in a metal jig and tested using a universal testing machine (ACME Engineers, Pune, India; Model No. UNITEST-10). UTM was equipped with a 10-kN load cell; crosshead speed: 0.5 mm/min. Shear force was applied parallel to the composite-substrate interface using a chisel-edge applicator (Figure 2).

**Figure 2:**
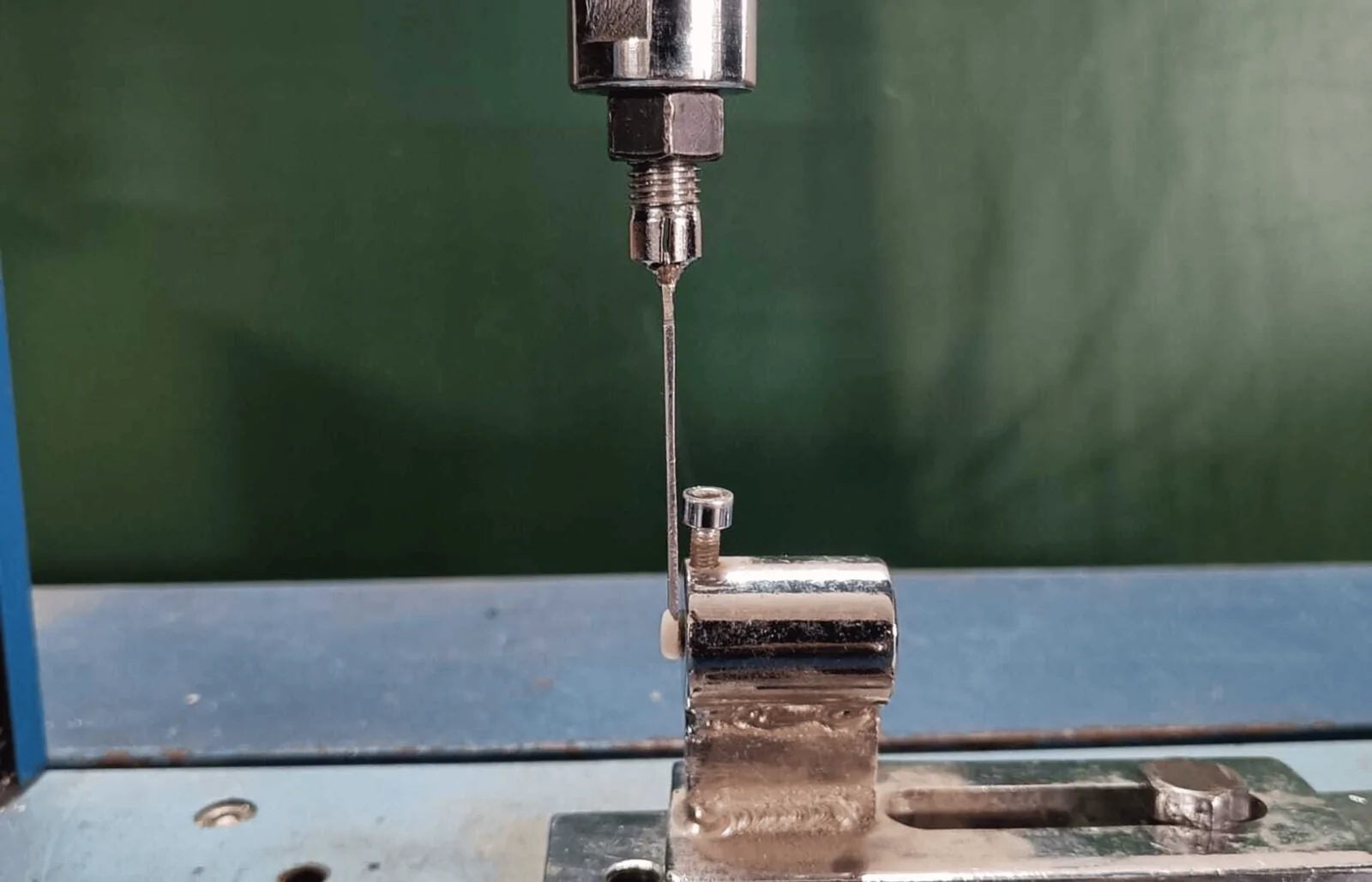
Shear bond strength measurement test using a universal testing machine* at a crosshead speed of 0.5 mm/minute ^*^ACME Engineers. Model No. UNITEST-10

Fracture loads in kilograms were recorded digitally and then converted into shear bond strengths measured in megapascals, as shear bond strength (MPA) = Fracture load (kg)/Area of the composite (mm²). Each specimen was examined under a stereoscopic lens ( Wuzhou New Found Instrument Co. Ltd, China Model: XTL 3400E, Magnification: 10 X) the images were captured using a computer-based program (Chroma Systems PVT. LTD., Model: MVIG 2005) the type of failure was assessed by one examiner who was properly trained and calibrated, and it was classified as adhesive (failure occurring at the interface between the substrate and the resin), cohesive (failure occurring within the substrate or the restoration material), or mixed (a combination of both adhesive and cohesive failures).

All statistical analyses were conducted using IBM SPSS statistical software, version 25 (IBM Corp., Armonk, NY). The descriptive analysis involved presenting the frequency and proportions of all explanatory and outcome variables for categorical data, and the mean and standard deviation (SD) for continuous data. Within-group comparison was done using one-way ANOVA followed by Tukey’s post hoc test. Because failure mode was categorical and several expected cell counts were small, the distribution of failure modes across the eight repair-system/surface-treatment combinations was evaluated using the Fisher-Freeman-Halton exact test.

## Results

Tables 4-5 present the findings of a one-way ANOVA comparing maximum load and shear bond strength across the four subgroups within Groups A and B, respectively. In Group A (Table 4), surface treatment with glycine powder demonstrated the highest mean maximum load and shear bond strength, although considerable variability was observed (SD = 253.005 N and 5.05 MPa, respectively). One-way ANOVA revealed a statistically significant difference among the subgroups for both maximum load (F = 3.490, p = 0.029) and shear bond strength (F = 3.244, p = 0.037), indicating that the type of surface treatment had a significant effect on these mechanical properties. In Group B (Table 5), glycine powder again exhibited the highest mean maximum load and shear bond strength.

**Table 4:** Comparison of maximum load and shear bond strength between four subgroups of group A* using one-way ANOVA followed by Tukey’s post hoc test ^*^Ivoclar repair system APF: acidulated phosphate fluoride; ANOVA: analysis of variance

Parameter	Group	Mean	Std. deviation	Std. error	95% confidence interval for mean		Minimum	Maximum	F Value	P-value
					Lower bound	Upper bound				
Max. load	Diamond bur	358.63	70.287	24.850	299.86	417.39	263	458	3.490	0.029
	Glycine powder	477.63	253.005	89.451	266.11	689.14	140	814		
	APF	365.25	148.204	52.398	241.35	489.15	158	570		
	Phosphoric acid	231.88	39.862	14.093	198.55	265.20	185	291		
Shear bond strength	Diamond bur	7.1725	1.39774	.49418	6.0040	8.3410	5.23	9.10	3.244	0.037
	Glycine powder	9.3363	5.05488	1.78717	5.1103	13.5622	2.77	16.18		
	APF	7.2800	2.91066	1.02907	4.8466	9.7134	3.25	11.30		
	Phosphoric acid	4.6300	0.80630	0.28507	3.9559	5.3041	3.60	5.79		

**Table 5:** Comparison of maximum load and shear bond strength between four subgroups of group B* using one-way ANOVA followed by Tukey’s post hoc test ^*^Shofu repair system APF: acidulated phosphate fluoride; ANOVA: analysis of variance

Parameter	Group	Mean	Std. deviation	Std. error	95% confidence interval for mean		Minimum	Maximum	F Value	P-value
					Lower bound	Upper bound				
Max. load	Diamond bur	270.38	69.705	24.645	212.10	328.65	190	386	3.457	0.030
	Glycine powder	303.50	39.330	13.905	270.62	336.38	245	365		
	APF	243.00	37.520	13.265	211.63	274.37	185	286		
	Phosphoric acid	237.63	25.145	8.890	216.60	258.65	201	270		
Shear bond strength	Diamond bur	5.3188	1.36279	0.48182	4.1794	6.4581	3.75	7.42	2.767	0.060
	Glycine powder	5.9500	0.83678	0.29585	5.2504	6.6496	4.42	6.98		
	APF	4.8888	0.75006	0.26518	4.2617	5.5158	3.75	5.68		
	Phosphoric acid	4.7487	0.50001	0.17678	4.3307	5.1668	4.00	5.30		

Table 6 summarizes the distribution of failure modes across the eight repair-system/surface-treatment combinations. Because the failure-mode variable is categorical and several expected frequencies are small, the Fisher-Freeman-Halton exact test was used instead of the chi-squared test. The exact two-sided p-value was 0.029, indicating a statistically significant association between repair-system/surface-treatment combination and failure mode at the 5% significance level. The observed distribution showed that cohesive failures were most frequent overall (31/64, 48.4%), followed by adhesive failures (25/64, 39.1%) and mixed failures (8/64, 12.5%).

**Table 6:** Distribution of failure modes according to repair system and surface treatment (Fisher-Freeman-Halton exact test) Values are presented as n/N (%). Percentages for individual experimental groups are calculated using N = 8 specimens per group, whereas percentages in the overall total row are calculated using N = 64 specimens. Exact p-value calculated using the Fisher-Freeman-Halton exact test for the 8 × 3 contingency table APF: acidulated phosphate fluoride

Repair system	Surface treatment	Cohesive, n (% of total)	Adhesive, n (% of total)	Mixed, n (% of total)	Total	Exact test	P-value
Ivoclar	Diamond bur	5 (7.8%)	3 (4.7%)	0 (0.0%)	8		
Ivoclar	Glycine powder	6 (9.4%)	2 (3.1%)	0 (0.0%)	8		
Ivoclar	APF	5 (7.8%)	1 (1.6%)	2 (3.1%)	8		
Ivoclar	Phosphoric acid	4 (6.3%)	3 (4.7%)	1 (1.6%)	8		
Shofu	Diamond bur	3 (4.7%)	5 (7.8%)	0 (0.0%)	8		
Shofu	Glycine powder	5 (7.8%)	1 (1.6%)	2 (3.1%)	8		
Shofu	APF	3 (4.7%)	5 (7.8%)	0 (0.0%)	8		
Shofu	Phosphoric acid	0 (0.0%)	5 (7.8%)	3 (4.7%)	8		
Total		31 (48.4%)	25 (39.1%)	8 (12.5%)	64	Fisher-Freeman-Halton exact test	0.029

The primary objective of this study was to evaluate the effects of different surface treatments such as diamond bur glycine powder, phosphoric acid, and APF gel on the maximum load, shear bond strength, and failure modes in intraoral repair of metal-ceramic restorations. The null hypothesis stated that the type of surface treatment would not significantly affect these mechanical and adhesive properties.

## Discussion

The primary purpose of this study was to assess the influence of various surface treatments (glycine powder, phosphoric acid, and APF gel) on the maximum load, shear bond strength, and failure modes in intraoral repair of metal-ceramic restorations. A shear test was selected for this study as it is clear from existing literature that anterior metal-ceramic restorations are more likely to fracture. These restorations are mainly exposed to shear stress, and the shear bond test is seen as a suitable method for measuring the strength of porcelain repairs within the mouth [12].

The data did not support rejecting the null hypothesis for maximum load and shear bond strength in all groups, as the p-values for some comparisons (especially in Group B for shear bond strength) were above the commonly accepted significance level of 0.05. However, notable trends emerged, particularly in Group A, where glycine powder exhibited the highest mean values for both maximum load (477.63 N) and shear bond strength (9.34 MPa), despite a high degree of variability (SD = 253.005 N and SD = 5.05 MPa).

These findings are consistent with earlier research, which found that air abrasion produced greater bond strength for intraoral repair systems when applied to ceramic and metal surfaces compared to using a diamond bur for surface roughening [9,27]. Gourav et al. (2016) also reported that surface treatment with sandblasting yielded the highest shear bond strength among the protocols tested, followed by the combination of sandblasting and acid etching. Their research examined how four different ways of preparing surfaces affected the strength of the bond between porcelain repair systems and the material, highlighting the significance of changing the surface mechanically to improve the effectiveness of the adhesive [28].

The observed differences in surface roughness and shear bond strength suggest that micromechanical retention may contribute to adhesion in metal-ceramic repair systems. These findings were further substantiated by SEM. These morphological differences closely corresponded to the surface roughness (Ra) values reported in Table 2, where glycine powder achieved the highest Ra values.

For Group B, glycine powder again demonstrated the highest mean maximum load (303.50 N) and shear bond strength (5.95 MPa), but the statistical significance for shear bond strength (p = 0.060) did not reach the threshold for acceptance. The significant difference in maximum load (p = 0.030) in Group B, compared to the absence of significant differences in shear bond strength, suggests that glycine powder may have a more pronounced effect on mechanical strength than on adhesive bond strength in some conditions.

The primary focus of the present study was to evaluate the effect of different surface-treatment modalities on the repair of ceramic surfaces using two commercially available repair systems. Although differences were observed between the two repair systems, these systems involved distinct repair protocols, including different primers, bonding agents, opaques, and composite materials. Therefore, any differences observed between the repair systems should be interpreted as differences in the performance of their respective complete repair protocols and should not be attributed solely to the repair-system designation. The principal interpretation of the present study remains focused on the effect of the surface-treatment modalities within the evaluated repair protocols. Further studies using standardized repair materials and bonding protocols would be required to isolate the independent contribution of individual repair-system components.

However, our study also found that phosphoric acid resulted in the lowest values for both maximum load and shear bond strength. These findings are in agreement with Pattanaik et al. (2011), who reported similarly low bond strength following surface treatment with 37% phosphoric acid. In their study, the control group without surface treatment exhibited the lowest mean shear bond strength, followed closely by the phosphoric acid-treated group, highlighting the limited efficacy of this agent in enhancing adhesion to metal-ceramic surfaces [29]. Failure-mode analysis demonstrated variation across the eight repair system-surface treatment combinations (Table 6). Cohesive failure was the most frequent failure mode overall (31/64, 48.4%), followed by adhesive failure (25/64, 39.1%) and mixed failure (8/64, 12.5%).

The Fisher-Freeman-Halton exact test demonstrated a statistically significant association between repair system-surface treatment combination and failure mode (p = 0.029), indicating that the distribution of failure modes was not homogeneous across the tested combinations. Although fewer adhesive failures were observed in some glycine powder- and APF-treated subgroups, the overall exact test does not establish that any individual surface treatment was superior in preventing adhesive failure. Therefore, these findings should be interpreted as evidence of an overall difference in failure mode distribution rather than as proof of superiority of a specific surface-treatment modality.

LImitations

Despite these findings, several limitations should be considered. The relatively small sample size of eight specimens per experimental condition may have limited the ability to detect smaller differences between treatment modalities. The high variability observed in Group A, particularly for glycine powder, suggests that experimental variability may have influenced the observed outcomes and warrants cautious interpretation. Additionally, the lack of statistical significance for shear bond strength in Group B indicates that a larger sample size may be required to detect smaller differences. Although the Fisher-Freeman-Halton exact test demonstrated a statistically significant overall association between the eight repair system/surface-treatment combinations and failure mode (p = 0.029), the small subgroup size (n = 8) limits the precision of individual subgroup estimates and precludes strong conclusions regarding specific pairwise differences in failure-mode distribution.

The absence of an untreated surface control and a conventional aluminum-oxide air-abrasion comparator limits the ability to compare the evaluated surface treatments with untreated surfaces or with a commonly used conventional air-abrasion method. Furthermore, only two commercially available repair systems were evaluated, and these systems use different proprietary primers, bonding agents, opaques, and composite materials. Therefore, differences observed between the repair protocols cannot be attributed solely to the repair-system designation, and the findings should not be generalized to ceramic repair systems more broadly.

The absence of individual specimen-level surface roughness measurements limited the analysis of Ra values to descriptive reporting and precluded reliable inferential statistical comparison among the treatment groups. In addition, detailed instrument-specific parameters and measurement documentation were not available in the retained experimental records. Therefore, the reported Ra values should be interpreted within the context of the present in vitro experimental conditions, and direct comparisons with studies using different surface-roughness measurement protocols should be made with caution.

Although thermocycling was incorporated before mechanical testing, the relatively limited thermocycling protocol and absence of mechanical fatigue testing restrict conclusions regarding long-term clinical durability. Future studies should incorporate extended aging protocols and mechanical fatigue testing to better evaluate the durability of repaired metal-ceramic restorations under simulated clinical conditions.

Future studies with larger sample sizes are recommended to more conclusively evaluate the influence of surface treatment and repair protocol on failure-mode distribution. Further research should also evaluate appropriate control and conventional comparator groups and investigate different surface-treatment concentrations, application times, bonding protocols, and environmental conditions. Overall, glycine powder showed the highest mean maximum load and shear bond strength in both repair systems; however, the statistical significance of these effects differed according to the repair system and outcome. Further studies are needed to determine the clinical relevance of these findings under simulated and clinical conditions.

## Conclusions

Within the limitations of this in vitro study, surface treatment was found to significantly influence both maximum load and shear bond strength in metal-ceramic repair systems. In Group A (Ivoclar system), statistically significant differences were observed for maximum load and shear bond strength, supporting the rejection of the null hypothesis for these mechanical parameters. Glycine powder demonstrated the highest mean values for both evaluated parameters in both repair-system groups, suggesting favorable performance as a surface-treatment modality under the conditions of the present study. Although the increase in shear bond strength in Group B (SHOFU system) did not reach statistical significance, a positive trend was observed. Phosphoric acid consistently resulted in the lowest maximum load and shear bond strength values, in agreement with previous studies reporting its limited effectiveness as a conditioning agent for metal-ceramic surfaces.

Failure mode analysis demonstrated a statistically significant overall association between the repair-system/surface-treatment combinations and failure mode. Cohesive failures were most frequent overall; however, because the Fisher-Freeman-Halton exact test evaluates the overall contingency table, it does not establish superiority of an individual treatment without additional adequately powered comparisons. The results of this study apply to the population and experimental conditions tested and underscore the importance of appropriate surface treatment in enhancing the mechanical and adhesive performance of intraoral repair systems. However, the observed variability, particularly in Group A, and the lack of statistical significance for certain outcomes highlight the need for further research with larger sample sizes and under simulated clinical conditions to validate and expand upon these findings.
